# Oxidative T Cell Modifications in Lupus and Sjogren’s Syndrome

**Published:** 2017-01-10

**Authors:** FM Strickland, T Mau, M O’Brien, A Ghosh, BC Richardson, R Yung

**Affiliations:** Department of Medicine, University of Michigan, Ann Arbor MI, USA

**Keywords:** Lupus, Sjogren’s syndrome, Oxidative stress, Nitration, T lymphocytes, Subsets

## Abstract

**Objectives:**

Lupus flares are triggered by environmental agents that cause oxidative stress, but the mechanisms involved are unclear. The flares are characterized by oxidative modifications of proteins by 4-hydroxynonenals, malondialdehydes, carbonyls and nitration. These modifications have been proposed to induce and perpetuate lupus flares by “altered self” mechanisms. An epigenetically altered CD4+CD28+ T cell subset, caused at least in part by nitration of T cell signaling molecules, is found in patients with active lupus, and nitrated T cells are sufficient to cause lupus-like autoimmunity in animal models. The relation of protein 4-hydroxynonenals, malondialdehydes, carbonyls and nitration to lupus flares though, is unknown. We tested if the size of the epigenetically altered subset is related to disease activity and one or more of these oxidative modifications in lupus patients. We also tested the relationship between subset size, disease activity and the same oxidative modifications in Sjogren’s syndrome, another autoimmune disease also associated with oxidative stress and characterized by anti-nuclear antibodies and the presence of the subset.

**Methods:**

Lupus flare severity was quantitated using the Systemic Lupus Erythematosus Disease Activity Index, and Sjogren’s flare severity using the European Sjogren’s Syndrome Disease Activity Index. Subset size was determined by flow cytometry. Protein modifications were determined by ELISA.

**Results:**

Only protein nitration correlated with the size of the subset in lupus and Sjogren’s syndrome.

**Conclusions:**

These results support a role for protein nitration in subset size and lupus flare severity. Protein nitration may also contribute to autoantibody formation in Sjogren’s syndrome.

## Introduction

Systemic lupus erythematosus (SLE) is a chronic, relapsing autoimmune disease characterized by autoantibodies to nuclear and other self-antigens, and requires both a genetic predisposition and an environmental exposure to develop and flare. The genes predisposing to lupus are being characterized. However, the mechanisms by which environmental agents trigger lupus flares are less well understood. Agents associated with lupus flares include those causing oxidative stress such as sun exposure, infections, silica and smoking, and lupus flares themselves generate oxidative stress [1]. Lupus-like autoantibodies such as anti-nuclear antibodies as well as anti-Sm, anti-Ro, anti-La and anti-cardiolipin, and occasionally clinical features of lupus, can also be found in patients with other chronic autoimmune diseases characterized by inflammation and oxidative stress such as Sjogren’s syndrome, rheumatoid arthritis, systemic sclerosis, and overlap syndromes [2–6]. The mechanisms by which oxidative stress initiate autoantibody formation and lupus flares though, are unclear.

Lupus flares are characterized by oxidative modifications of proteins with 4-hydroxynonenal (HNE), malondialdehyde (MDA) and carbonyls, which have been proposed to contribute to lupus flares through mechanisms including altered self, promoting antigenicity and formation of autoantibodies to the novel epitopes [7]. Protein nitration, caused by peroxynitrite (ONOO−) covalently binding tyrosines to form 3-nitrotyrosine, also correlates with lupus flares [7].

T cell DNA demethylation may also contribute to lupus flares. DNA methylation refers to the methylation of dC bases in CpG pairs and is a repressive modification. DNA methylation patterns are established during development and silence genes unnecessary for the function of any given cell. The patterns are then replicated each time a cell divides by DNA methyltransferase 1 (Dnmt1). Inhibiting CD4+ T cell Dnmt1 during mitosis prevents methylation of newly synthesized DNA in the daughter cells, causing demethylation and overexpression of genes that convert normal, antigen specific “helper” T cells into autoreactive, proinflammatory, cytotoxic cells that are sufficient to cause lupus-like autoimmunity in murine models [8]. Importantly, procainamide and hydralazine are drugs that cause lupus-like autoimmunity in genetically predisposed people, and both inhibit T cell DNA methylation and alter gene expression. Further, CD4^+^ T cells treated with these drugs or other DNA methylation inhibitors such as 5-azacytidine become autoreactive and cause lupus-like anti-DNA antibodies in non-lupus prone mice, and anti-DNA antibodies and immune complex mediated glomerulonephritis in lupus-prone mice [8]. Patients with active lupus also have similar hypomethylated, autoreactive CD4^+^ T cells which comprise a novel subset, the size of which is directly related to disease activity [9], suggesting that these T cells may participate in flares of human lupus as they do in the murine models. How this epigenetically altered T cell subset develops is unclear.

We traced the lupus T cell DNA methylation defect to a failure to upregulate DNA methyltransferase 1 (Dnmt1) as the cells enter mitosis. The signaling defect was traced to PKCδ, which is catalytically inactive in T cells from patients with active lupus, and the inactive fraction was found to be nitrated [10], implicating ONOO− mediated protein nitration as a mechanism contributing to PKCδ inactivation and lupus flares. We then tested the effects of protein nitration on T cell gene expression by treating PHA stimulated CD4^+^ T cells with ONOO− using protocols previously used to inhibit DNA methylation in T cells, and found that both DNA methylation inhibitors and ONOO^−^ increase methylation sensitive gene expression [11]. However, the relative contributions of HNE, MDA, carbonyls and protein nitration to subset size and lupus flare severity are unclear.

We hypothesized that levels of oxidative modifications that contribute to disease activity in SLE would be proportional to subset size. We therefore compared the size of the subset to serum 3NT, PC, HNE and MDA levels, and to disease activity, in patients with inactive and active lupus. We also compared subset size, disease activity and 3NT, PC, HNE and MDA levels in patients with SS, a related autoimmune disease also characterized by oxidative stress [12], the presence of the subset and lupus-like autoantibodies [9].

## Methods

### Subjects

Women with primary SS ages 24–79 and SLE ages 21–66 were recruited from the outpatient clinics at the University of Michigan. Patients receiving cyclophosphamide were excluded because of effects on cell surface markers [13] and methotrexate because of effects on DNA methylation [14]. The other medications commonly used to treat SLE and SS, including corticosteroids, hydroxychloroquine and antimetabolites like azathioprine, have not been found to affect T cell DNA methylation [8]. Patients with lupus met the revised criteria for the classification of lupus [15] and patients with SS met the American College Classification Criteria for Sjogren’s syndrome [16]. Lupus disease activity was measured using the Systemic Lupus Erythematosus Disease Activity Index (SLEDAI) (Table 1) [17] and SS activity using the 2002 EULAR Sjogren’s Syndrome Disease Activity Index (ESSDAI) (Table 2) [18]. This study was approved by the University of Michigan Institutional Review Board.

### Flow cytometric analyses

Peripheral blood mononuclear cells (PBMC) from patients with lupus and SS were stained with fluorochrome conjugated monoclonal antibodies to CD3, CD4, CD28, CD70, CD40L, and KIR proteins then analyzed by multicolor flow cytometry as described [9]. Results are expressed as the percent CD3^+^CD4^+^CD28^+^CD70^+^CD40LhiKIR^+^ T cells relative to total CD3^+^CD4^+^CD28^+^ T cells.

### Protein oxidative modifications

Serum protein 3-nitrotyrosine, carbonyl, MDA and HNE levels were measured using ELISA kits from Cell Biolabs, San Diego CA.

### Statistical analysis

The relationship between serum protein modifications and T cell subset size was determined by linear regression using Prism 6 software from GraphPad (San Diego CA).

## Results

**Figure 1:**
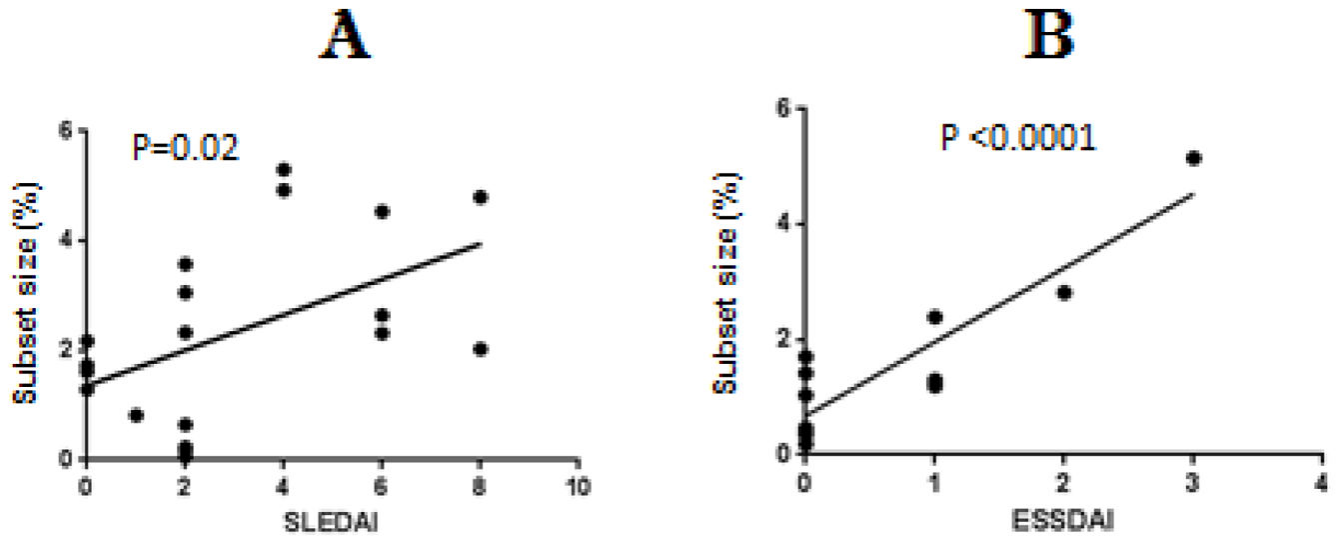
Disease activity and subset size. PBMC from 18 patients with inactive and active lupus or 9 patients with Sjogren’s Syndrome were stained with fluorochrome conjugated monoclonal antibodies to CD3, CD4, CD28, CD11a, CD70, CD40L, and KIR proteins, then the size of the epigenetically altered CD3+CD4+CD28+CD70+CD40L+KIR+ subset relative to total CD3+CD4+CD28+ T cells was measured by multicolor flow cytometry. Subset size was plotted against (A) lupus disease activity as measured by the SLEDAI or (B) Sjogren’s disease activity as measured by the ESSADAI. Significance of the relationship between subset size and disease activity was tested by linear regression.

Initial studies compared the size of the epigenetically altered T cell subset to disease activity in patients with lupus and SS. Lupus disease activity was quantified using the SLEDAI [17] and SS activity with the ESSDAI [18]. PBMC from women with inactive and active lupus and inactive and active SS were isolated and stained with fluorochrome conjugated antibodies to CD3, CD4, CD28, CD70, CD40L and the KIR gene family then analyzed by multicolor flow cytometry [9]. Figure 1A shows that the size of the epigenetically altered subset increases in direct relation to the SLEDAI as previously described by our group [9]. Figure 1B shows that the subset is also found in patients with SS, and that the size of the subset also increases with disease activity as measured by the ESSDAI.

**Figure 2:**
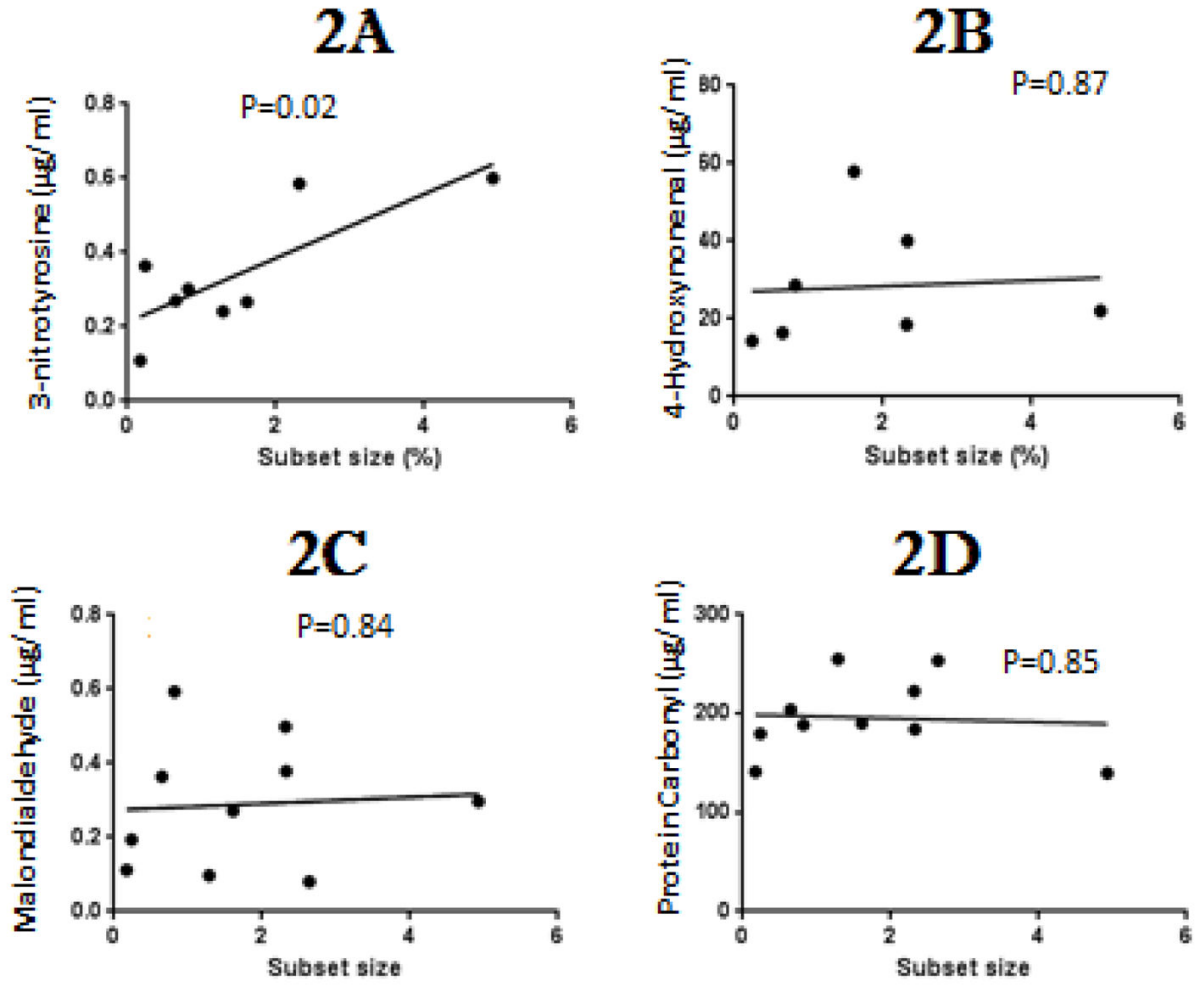
Relationship between subset size and biomarkers of oxidative stress in Lupus patients. Levels of 2A- 3-nitrotyrosine, 2B- 4-hydroxynonenal, 2C- malondialdehyde and 2D- carbonyls were measured in serum proteins from patients with inactive and active Sjogren’s and plotted against subset size. The relationship between subset size and serum protein modification levels was tested by linear regression.

**Figure 3:**
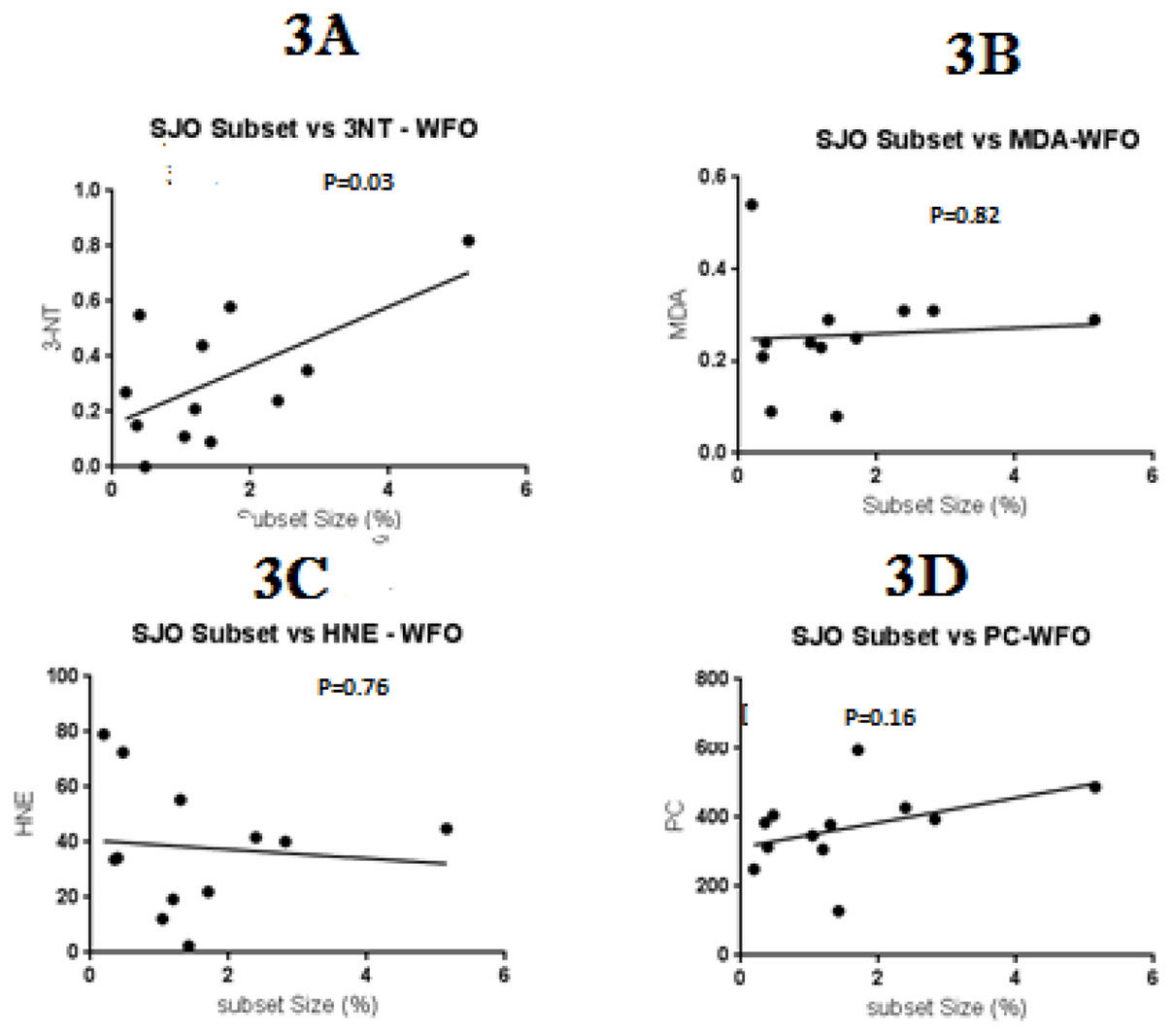
Relationship between subset size and biomarkers of oxidative stress in Sjogren’s Syndrome patients. Levels of 3A- 3-nitrotyrosine, 3B- 4-hydroxynonenal, 3C- malondialdehyde and 3D- carbonyls were measured in serum proteins from patients with inactive and active Sjogren’s and plotted against subset size. The relationship between subset size and serum protein modification levels was similarly tested by linear regression.

As noted above, lupus flares are triggered by agents causing oxidative stress, and the flares themselves cause further inflammation and oxidative stress, resulting in oxidative modifications of serum proteins including the formation of 3-NT [8], as well as HNE, MDA and PC adducts [7]. We therefore tested if the level of these protein modifications are directly related to the size of the epigenetically altered subset associated with the flares. Figure 2A shows that serum protein 3-NT levels are directly proportional to the size of the subset, consistent with a model in which T cell PKCδ inactivation by nitration initiates development of the epigenetically altered T cell subset. In contrast, there was no significant relationship between subset size and levels of HNE (Figure 2B), MDA (Figure 2C) or PC (Figure 2D) modified proteins. Since a similar subset is found in patients with SS [9], also characterized by biomarkers of oxidative stress [12], we tested whether the subset size was also proportional to 3-NT, HNE, MDA and PC levels in this disease. Figure 3 shows that the subset size again is significantly related to 3-NT but not HNE, MDA or PC, further supporting a role for protein nitration but not MDA, HNE or PC in development of the epigenetically altered cells.

Together, these results support a model in which oxidative stress contributes to the development of autoantibodies in lupus, Sjogren’s Syndrome and possibly other inflammatory diseases by inactivating T cell PKCδ through nitration, decreasing ERK pathway signaling and Dnmt1 levels, epigenetically altering gene expression in a subset of CD4^+^ T cells.

## Discussion

A body of literature supports the concept that environmental agents which cause oxidative stress, such as sun exposure, infections, silica and smoking [1], are associated with lupus onset and flares, but the cells and mechanisms involved are unclear. Drugs such as hydralazine and procainamide are also associated with lupus-like autoimmunity [8] and have provided insights into these mechanisms. Earlier studies demonstrated that inhibiting DNA methylation in antigen specific CD4^+^ T cells with DNA methyltransferase inhibitors like 5-azacytidine increases expression of genes normally silenced by DNA methylation, making the cells autoreactive, and that the autoreactive cells cause lupus-like autoimmunity in animal models [8]. These studies also demonstrated that procainamide and hydralazine are DNA methylation inhibitors, and that T cells from patients with active lupus have hypomethylated DNA, overexpress the same genes as CD4^+^ T cells treated with 5-azacytidine, procainamide or hydralazine, and are similarly autoreactive [8].

Array based genome scanning approaches demonstrated that the epigenetically modified T cells overexpress genes including ITGAL (CD11a), which contributes to the autoreactivity, TNFSF7 (CD70) and CD40LG (CD40L) which contribute to B cell overstimulation, and the KIR gene family, contributing to interferon gamma overexpression and macrophage killing [8]. However, whether these genes are expressed on the same cell, or if different cells express a different repertoire of the genes was unclear. Subsequent studies used multicolor flow cytometry to demonstrate that these genes are co-overexpressed on the same CD3^+^CD4^+^CD28^+^ T cells, representing a previously unrecognized T cell subset, and that the size of this subset is directly related to lupus flare severity [9]. However, the mechanisms by which environmentally induced oxidative stress alters T cell gene expression were unclear.

Other reports demonstrated that the oxidative stress associated with lupus flares is manifested in part by the nitration of protein tyrosines (Tyr), forming 3-nitroTyr, and that serum levels of nitrated proteins are directly related to disease activity in lupus patients [7]. The nitration is mediated by peroxynitrite (ONOO^−^), formed by superoxide (O_2_^−^), generated during inflammatory responses, combining with nitric oxide (NO), an intracellular signaling molecule, to form ONOO−. We therefore compared the effects of H_2_O_2_ and ONOO^−^ on CD4^+^ T cell gene expression in vitro and in vivo. These studies revealed that treating human CD4^+^ T cells with H_2_O_2_ or ONOO^−^ decreased Dnmt1 levels, causing demethylation and overexpression of genes demethylated and overexpressed in CD4^+^ T cells from patients with active lupus, and that ONOO^−^ was more potent than H_2_O_2_, likely reflecting a requirement for O_2_^−^ to combine with NO to produce ONOO^−^ [10]. Other studies demonstrated that murine CD4^+^ T cells treated with H_2_O_2_ or ONOO^−^ cause a lupus-like disease when injected into SJL mice [11]. As noted above, earlier studies also traced the signaling defect to PKCδ, which was found to be catalytically inactivated by nitration both in T cells oxidized in vitro and in T cells from patients with active lupus [10]. The importance of T cell PKCδ inactivation in lupus pathogenesis was confirmed by creating a double transgenic mouse strain in which expression of a dominant negative PKCδ (dnPKCδ) is selectively activated in CD4^+^ T cells by adding doxycycline to their drinking water. Inducing dnPKCδ expression decreased ERK pathway signaling, decreased Dnmt1 levels, and caused overexpression of CD70 (TNFSF7) normally silenced by DNA methylation in T cells. These mice also developed anti-DNA antibodies and an immune complex mediated glomerulonephritis [8].

The significance of this subset in patients with SS, as well as patients with rheumatoid arthritis and progressive systemic sclerosis [9], is less clear. Inflammation caused by the autoimmune disease process in these disorders may generate the subset, which could contribute to development of the anti-nuclear antibodies seen in these diseases. This hypothesis is supported by reports that T cells responding to host MHC molecules following hematopoietic stem cell transplantation can cause features of autoimmune diseases including lupus, systemic sclerosis, and others in humans [19].

Together these studies indicate that oxidative stress may contribute to lupus flares through mechanisms including protein nitration. The results support a model in which nitration of T cell proteins including PKCδ leads to decreased ERK pathway signaling, decreased Dnmt1 levels and overexpression of methylation-sensitive genes that convert antigen specific T cells into autoreactive cells that contribute to lupus-like autoimmunity in genetically predisposed hosts [8]. These results also indicate a mechanism by which anti-oxidants may help prevent lupus flares.

## Figures and Tables

**Table 1:** Subjects for Systemic Lupus Erythematosus Disease Activity Index (SLEDAI).

Disease	Age	SLEDAI	Medications
Lupus	32	6	Hcq
Lupus	36	2	Hcq
Lupus	66	0	-
Lupus	57	4	MMF
Lupus	38	0	Hcq
Lupus	46	2	Hcq
Lupus	32	2	Hcq
Lupus	37	0	Aza
Lupus	39	1	Hcq
Lupus	37	1	Hcq
Lupus	36	6	Hcq, Quin
Lupus	49	8	Hcq, Quin
Lupus	23	0	Hcq
Lupus	28	4	-
Lupus	41	4	MMF, Hcq
Lupus	56	2	Hcq
Lupus	18	4	Quin, tofacitinib

**Table 2:** Subjects for EULAR Sjogren’s Syndrome Disease Activity Index (ESSDAI).

Disease	Age	ESSDAI	Medications
Sjogren’s	74	0	Hcq
Sjogren’s	56	0	Cevimiline
Sjogren’s	72	3	MMF
Sjogren’s	57	0	
Sjogren’s	53	1	
Sjogren’s	64	0	
Sjogren’s	60	0	
Sjogren’s	24	1	
Sjogren’s	56	1	Hcq

Note: Hcq: Hydroxychloroquine; MMF: Mycophenolate Mofetil; Quin: Quinacrine

## Abbreviations

KIR: Killer Cell Immunoglobulin-like Receptor
ORF: Open Reading Frame
HNE: Hydroxynonenal
MDA: Malondialdehyde
SLEDAI: Systemic Lupus Erythematosus Disease Activity Index
ESSDAI: Eular Sjogren’s Syndrome Disease Activity Index
